# The Long-Term Use of Communication Robots by Users of Visiting Healthcare Services: Development of an Integrated Vital Signs Measurement System

**DOI:** 10.7759/cureus.77635

**Published:** 2025-01-18

**Authors:** Toshiaki Takahashi, Shiho Higashimura, Tsuneki Ninomiya, Sachiyo Fujimura, Naoki Sugimoto, Tetsuhiko Horai, Tomoko Abe, Aya Kitamura, Masaru Matsumoto, Gojiro Nakagami, Hiromi Sanada

**Affiliations:** 1 Nursing, The University of Tokyo, Tokyo, JPN; 2 Nursing, Shonan University of Medical Sciences, Kamakura, JPN; 3 Robotics, Fujisoft Incorporated, Yokohama, JPN; 4 Rehabilitation, Visiting Nursing Station Kesera, Tokyo, JPN; 5 Nursing, Visiting Nursing Station Kesera, Tokyo, JPN; 6 Nursing, Ishikawa Prefectural Nursing University, Ishikawa, JPN

**Keywords:** communication robot, gerontological nursing, home heath care, older aged people, self care

## Abstract

Purpose

This study explores the use of communication robots (CRs) to sustain self-care behaviors among older adults, addressing motivational challenges.

Method

We used the CR PALRO® (FUJISOFT, Kanagawa, Japan) and developed a system to wirelessly transmit vital signs data (temperature, blood pressure, pulse oximetry) to cloud storage. PALRO reviewed the data and provided feedback to the user. One participant, a woman in her 90s living alone and using a nursing service, was recruited with ethical approval from the University of Tokyo's Ethical Review Board.

Results

Before using the CR, the participant had stopped measuring her temperature and blood pressure. After implementation, she measured her vital signs for 334 days, storing the data in the cloud. The system prompted measurements without reminders 248 times in 334 days (74.3%) and gave feedback when measurements were missed.

Conclusion/implications

The CR system is feasible for visiting care service users, promoting self-care and early abnormality detection. Future improvements could enhance self-care for independent older adults.

## Introduction

Maintaining health through proactive self-care is crucial, particularly for older adults and those who use visiting care services. Regular monitoring of vital signs such as blood pressure, temperature, and oxygen saturation plays a significant role in preventive healthcare [1,2]. These practices facilitate the early detection of potential health issues and the timely administration of appropriate interventions. However, it can be challenging to maintain these behaviors long-term. Individual motivation and active participation are essential for this purpose, yet they often fluctuate, -which can lead to irregular monitoring and potential neglect of health maintenance [3,4]. To address this, nurse-led proactive self-care programs can positively affect the psychological outcomes of independent, non-frail, community-dwelling older adults, thus highlighting the importance of professional support for maintaining health-sustaining behaviors [5].

In recent years, technological advancements have opened new avenues that can support self-care practices among older adults [6,7]. Communication robots (CRs), which provide interactive entertainment through conversations, singing, and other activities, have emerged as promising tools in this regard [8-10]. These robots not only engage users in enjoyable activities but also have the potential to encourage regular health monitoring [11,12]. The integration of CRs into daily life represents an innovative approach to addressing the lack of motivation often observed in this demographic in terms of maintaining traditional self-care practices.

This study aimed to investigate the feasibility of integrating vital signs measurement devices with CRs to promote sustained self-care behaviors among older adults. By developing a system that combines the interactive features of CRs with the practical utility of health monitoring devices, we sought to create a supportive environment that motivates and encourages older adults to engage in regular health monitoring. The ultimate goal was to improve the quality of life and health-related outcomes in this population by leveraging new technologies to enhance self-care practices.

## Materials and methods

Selection and integration of communication robots

**Figure 1:**
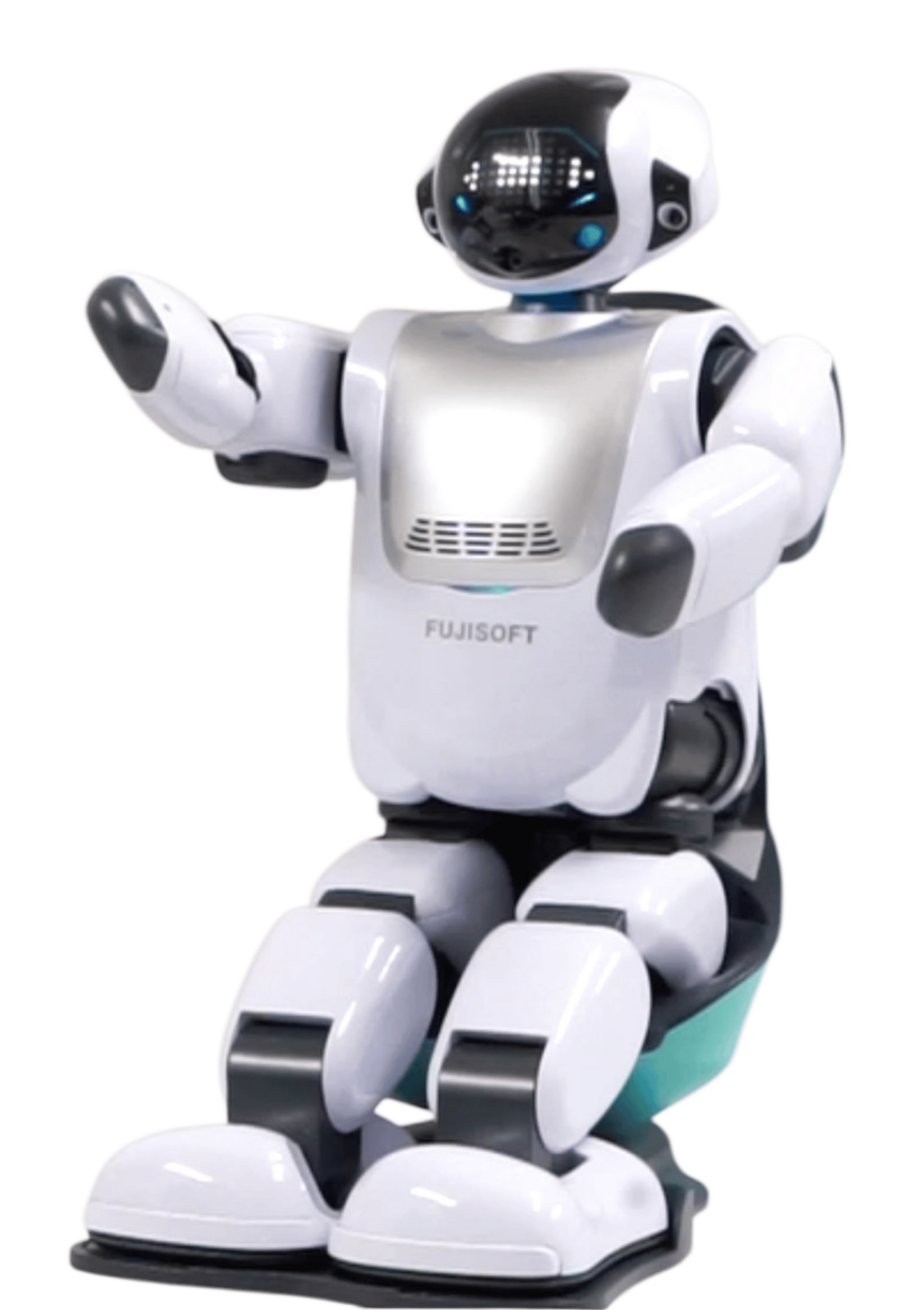
Communication robot PALRO® Image credits: Toshiaki Takahashi

We used PALRO® (FUJISOFT, Kanagawa, Japan), a commercially accessible communication robot (CR) designed for interactive engagement and health support. PALRO is equipped with advanced features, including voice recognition, speech synthesis, facial recognition, and the ability to perform various programmed activities, such as playing music, dancing, and storytelling (Figure 1). These features make it particularly effective in promoting cognitive stimulation and social interaction among older adults.

PALRO measures approximately 39 cm in height and weighs 1.5 kg, making it portable and suitable for bedside use. It is powered by rechargeable batteries, allowing for several hours of continuous operation. The robot operates via an embedded artificial intelligence system, which supports autonomous decision-making and personalized interactions based on user preferences and responses.

Previous studies have demonstrated PALRO's effectiveness in fostering physical activity, cognitive engagement, and social interactions among older adults in residential and group settings [13-15]. In our study, PALRO was strategically placed at the participant's bedside to function as both a health-monitoring tool and a source of social engagement, enhancing its utility in a real-world context.

System development and data management

This system focuses on seamless integration and user-friendly interaction. The vital signs and environmental data collected by the measuring devices were transmitted wirelessly to secure cloud storage through a small Raspberry Pi 3 Model B PC (RS Components, Corby, United Kingdom) to ensure data privacy and security. A Thermo Phrase MT-500BT thermometer (Nihon Seimitsu Sokki, Shibukawa, Japan), WSK-1021J blood pressure meter (Nihon Seimitsu Sokki, Shibukawa, Japan), pulse fit BO-750BT transcutaneous device for measuring the partial pressure of oxygen (Nihon Seimitsu Sokki, Shibukawa, Japan), and SwitchBot Meter Plus thermo-hygrometer (SwitchBot Global, Shenzhen, China) were used in this study. PALRO® was programmed to access this data periodically, analyze it, and provide users with personalized feedback and reminders, thus fostering a proactive approach to health monitoring. The subject's measurement data were provided in a form that could be viewed through a browser at the home nursing station that was caring for the subject.

User interaction and feedback mechanism

The system was designed to recognize the user's face and prompt them to take their measurements. Upon completion, PALRO® provided positive feedback, thanking the user for their cooperation. In cases where measurements had not been taken, the robot issued reminders, encouraging the user to perform the necessary health checks. This interaction was central to engaging the user and promoting regular self-care behaviors.

Study implementation and participant recruitment

The subject for this study was recruited from a pool of older adults who were receiving home care services through a visiting care service. The introduction of the CR system into the subject's home was conducted with utmost concern for both safety and usability.

Data analysis

We conducted a descriptive analysis of the data. Vital sign measurements taken autonomously by the subject prior to 15:00 were designated as "self-measured". If any missing measurements were taken after prompting by the robot, they were classified as "reminders". Any measurements received within 30 minutes following a reminder were categorized as "prompt-success". Measurements detected ≥30 min after a reminder were coded as "prompt-failure", whereas the absence of any detected measurement following a reminder was labeled as "measurement failure".

Ethical considerations

This study was approved by the Research Ethics Committee of the Faculty of Medicine, University of Tokyo (approval no.: 2020302NI-(1)).

## Results

Our study subject was a woman in her 90s who was living alone and was classified as care level 2. Before the introduction of the CR, she had discontinued the practice of measuring her temperature and blood pressure. The CR system was installed in her home in February 2021 by a research nurse and her regular visitation staff to ensure safety. Throughout the 334-day study period, vital sign measurements were continuously taken, and the data were systematically stored in the cloud.

The results revealed that, of the total vital sign measurements, "self-measured" instances completed before 15:00 occurred 248 times, accounting for 74.2% of the measurements. Missing measurements where the robot issued a "reminder" occurred 86 times (25.8%).

**Figure 2:**
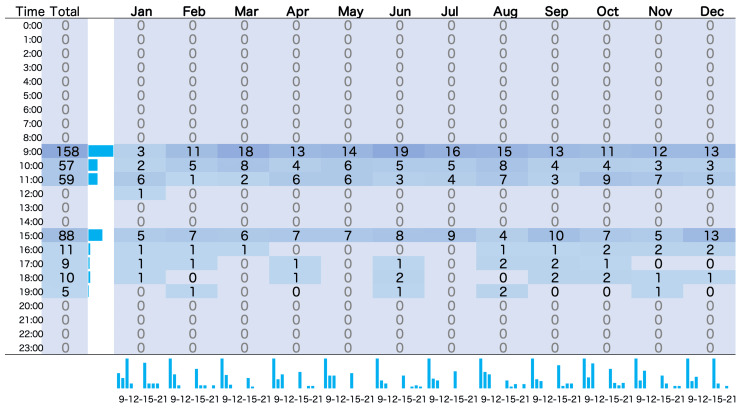
Heatmap of the frequency of the obtained measurements classified by hours

Further analysis indicated that among the reminders, "prompt-successes" (i.e., measurements taken within 30 minutes after a reminder) occurred 70 times (81.3%). "Prompt failures" (where measurements were detected after the 30-minute post-reminder window) were recorded 15 times (17.6%). There was one instance (1.1%) of "measurement failure" where no measurement was detected even after 30 minutes following a reminder. The reason for this measurement failure is unknown. The frequency of the obtained measurements is illustrated in a heat map categorized by hourly intervals (Figure 2). The data show that the majority of measurements were recorded in the morning, with the highest frequency occurring at 9:00 AM (158 measurements). Measurement frequencies were lower in the afternoon and evening, with few or no measurements recorded between 12:00 PM and 8:00 AM. A smaller increase in frequency was observed at 3:00 PM.

**Figure 3:**
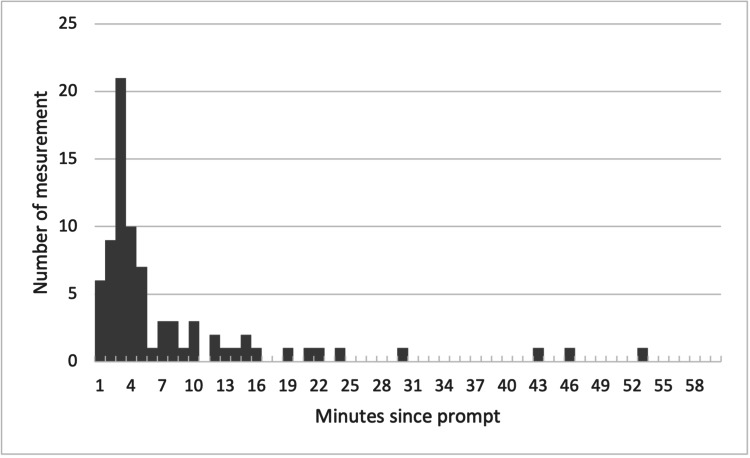
Histogram of elapsed time from prompt to measurement

**Figure 4:**
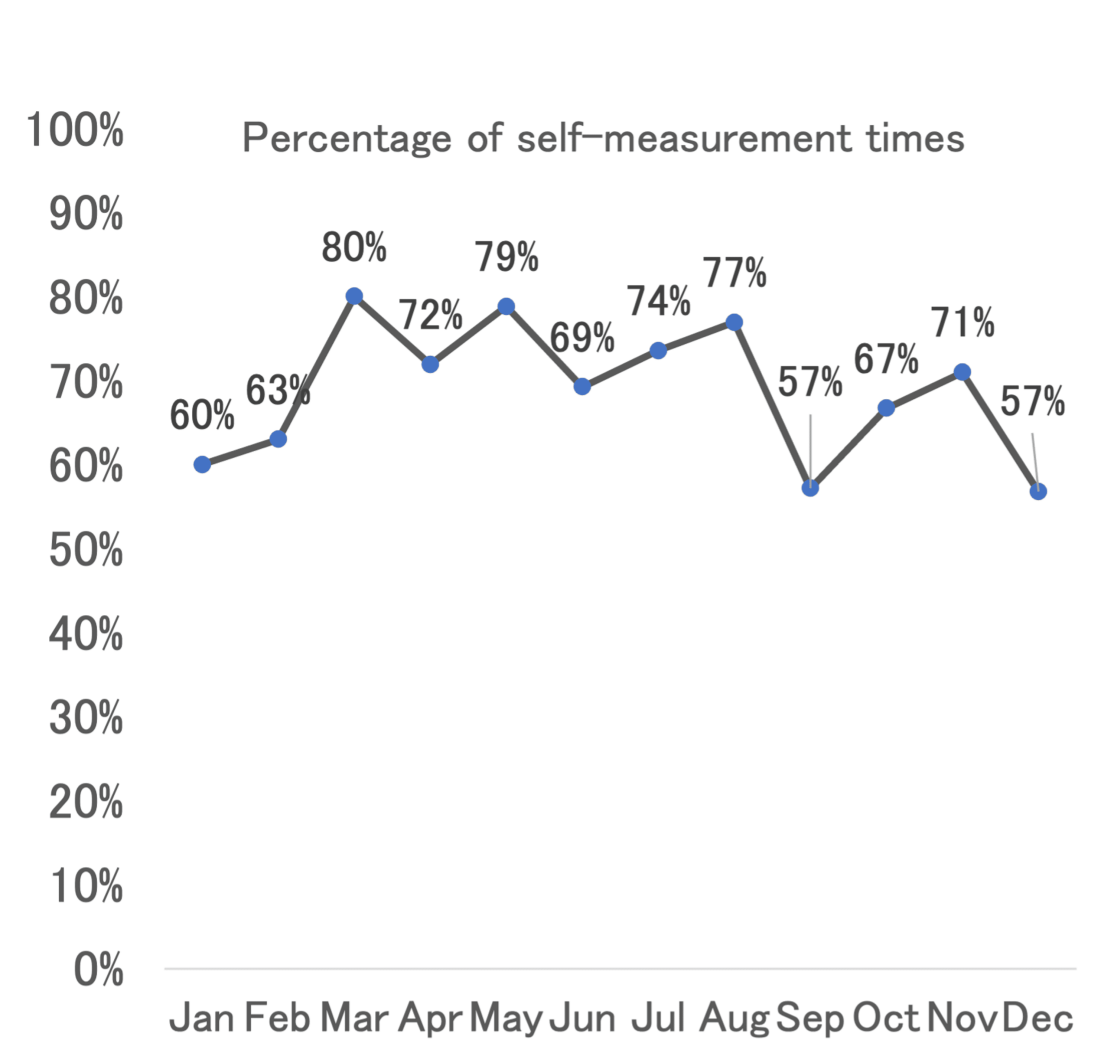
The percentage of "self-measured" instances by month

The average time from prompting to the execution of a measurement was seven minutes, with the system requiring three minutes to detect a measurement following a prompt. The times that elapsed from prompts to recorded measurements are shown in the histogram (Figure 3). The proportion of successful measurements maintained a roughly consistent trend across all periods, with no observed seasonal variations. The percentage of "self-measured" instances is presented by month in Figure 4. A comparison of the self-measurement percentages between March and August and the other months revealed a significant difference. The mean percentage of self-measurements for March to August was 75.2% ± 4.3%, while for the other months, it was 62.5% ± 5.6% (p=0.0016).

Notably, the system also facilitated early interventions by visiting nurses when high room temperatures were continuously recorded during the summer months, showcasing the potential for CRs in terms of monitoring and responding to environmental conditions as well.

## Discussion

This study demonstrated the potential of a CR integrated with a vital signs measurement system in terms of supporting sustained self-care behaviors among older adults. The system's ability to provide regular feedback and reminders significantly contributed to our subject's engagement with health monitoring activities. This finding underscores the importance of interactive technology for enhancing self-care practices and suggests a new direction related to caring for older adults.

The difference in self-measurement percentages may be explained by several factors. As the system was introduced in January, it is possible that the participant had not yet established a habit of self-measurement during the initial months. The higher percentages observed from March to August suggest that self-measurement was maintained at a high level for approximately six months. However, the decrease observed from September onwards could be attributed to habituation to the reminder announcements, potentially reducing their effectiveness over time. Despite this decline, the system successfully sustained measurement activity throughout the day, even if reminders were needed. This indicates that the system facilitated sufficient behavioral change, ensuring that measurements were performed consistently, including during the afternoon. The positive outcomes of this study will hopefully pave the way for further research focused on the integration of technology with healthcare practices. Expanding the system to include a wider array of sensing devices, as well as incorporating more sophisticated feedback mechanisms, may further enhance the effectiveness of such interventions. Additionally, integrating the control of home appliances such as air conditioners to respond to health-related data may offer a more holistic approach to supporting the well-being of older adults living independently. With personalized censoring and data storage, concepts such as the digital twin may be achievable in the near future [6,14].

However, it is often difficult to change the health-related behaviors of older adults themselves, even when predictions can be made using digital twins [14,15]. This study successfully promoted the adoption of new health-related behaviors in an older individual by using humanoid CR as a feedback interface, which represented a novel approach for the subject. As indicated by prior research, the significance of using humanoid robots, particularly ones capable of greeting users, might be significant compared to reminders delivered through computer graphics or onscreen displays [16,17].

This study provides preliminary insights into the feasibility of using a CR system for health monitoring. However, the lack of a control group limits the ability to evaluate the intervention's effectiveness. Future studies should incorporate control groups to enable a more rigorous assessment of the system's impact. Additionally, the small sample size and specific participant context limit the generalizability of the findings. Expanding future research to include larger and more diverse populations will enhance the system's applicability.

Finally, as the participant remained healthy throughout the study period, the system's performance during adverse health events could not be evaluated. Future studies should address these limitations to provide a more comprehensive understanding of the system's reliability and long-term utility in diverse healthcare settings.

## Conclusions

Our findings underscore the practicality and advantages of using CRs equipped with vital sign measurement devices to foster and maintain self-care behaviors among older adults. With ongoing technological advancements, the integration of such technologies into healthcare practices has emerged as a promising strategy to enhance the quality of life in older adults, empowering them to manage their health in an engaging and supportive manner.
